# Biofabrication of natural hydrogels for cardiac, neural, and bone Tissue engineering Applications

**DOI:** 10.1016/j.bioactmat.2021.03.040

**Published:** 2021-04-15

**Authors:** Kamil Elkhoury, Margaretha Morsink, Laura Sanchez-Gonzalez, Cyril Kahn, Ali Tamayol, Elmira Arab-Tehrany

**Affiliations:** a LIBio, Université de Lorraine, Nancy, F-54000, France; b Department of Applied Stem Cell Technologies, TechMed Centre, University of Twente, Enschede, 7500AE, the Netherlands; c Department of Biomedical Engineering, University of Connecticut, Farmington, CT, 06030, USA

**Keywords:** Hydrogel, Microfabrication, Bioprinting, Textiles, Tissue engineering, Regenerative medicine

## Abstract

Natural hydrogels are one of the most promising biomaterials for tissue engineering applications, due to their biocompatibility, biodegradability, and extracellular matrix mimicking ability. To surpass the limitations of conventional fabrication techniques and to recapitulate the complex architecture of native tissue structure, natural hydrogels are being constructed using novel biofabrication strategies, such as textile techniques and three-dimensional bioprinting. These innovative techniques play an enormous role in the development of advanced scaffolds for various tissue engineering applications. The progress, advantages, and shortcomings of the emerging biofabrication techniques are highlighted in this review. Additionally, the novel applications of biofabricated natural hydrogels in cardiac, neural, and bone tissue engineering are discussed as well.

## Highlights

• Different biofabrication techniques of natural hydrogels were overviewed.
• The progress, advantages, and disadvantages of the novel biofabrication strategies were discussed.
• Novel applications of biofabricated natural hydrogels in cardiac, neural, and bone tissue engineering were presented..
• Overlooked challenges facing the translation of biofabricated scaffolds and their potential solutions were highlighted.

## Graphical abstract

**Figure:**
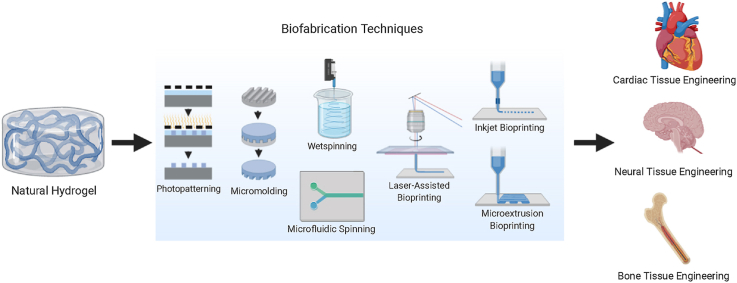

## 1 Introduction

Tissue engineering, as defined by Langer and Vacanti in 1993, is an interdisciplinary field that applies both the principles of life sciences and engineering to develop biological substitutes or entire organs [1]. Beyond that initial goal, tissue-engineered constructs have found a variety of new applications, such as being research tools that could improve our understanding and testing of diseases [[2], [3], [4], [5], [6]]. Furthermore, the development of personalized therapies is expected to be further facilitated by utilizing patient-specific cells and biological factors [[7], [8], [9], [10]]. Recently, tissue engineered constructs have even explored as tools for food production.

Hydrogels are high water content materials and one of the few biomaterials that can be used to fabricate extracellular matrix (ECM) mimicking scaffolds [11,12]. Moreover, in addition to being highly biocompatible, hydrogels possess an advantageous physical and biological tunability, and desirable robustness in biofabrication [[13], [14], [15]]. Although some synthetic materials have been shown to be less prone to evoke an immune response [16], naturally-derived crosslinked polymeric networks are preferred to avoid the potential risk of inflammatory and immunological responses induced by synthetic polymeric materials [[17], [18], [19], [20], [21], [22]]. However, the natural hydrogels can exhibit low mechanical properties and batch-to-batch variations due to their natural origin, which could limit their use [23].

Even though new types of tissues and organoids are being developed and fabricated, many challenges remain to be addressed before being able to create fully-functional tissues [24]. One of the main challenges is to create load-bearing structures that can replicate the complex architecture and physical properties of the native ECM. These type of structures cannot be fabricated using conventional techniques only, such as solvent casting/particulate leaching, freeze-drying, and gas foaming, but requires advanced biofabrication techniques, such as bioprinting and textile-based techniques [25,26]. Biofabricated textiles can form stronger supporting structures, whereas bioprinted constructs have more complex and controlled architectures [27].

These advances in biofabrication techniques have led to various novel applications in tissue engineering, from creating electro-active scaffolds, that modulate cell proliferation and differentiation, to smart scaffolds, that sustain the dynamic nature of the tissue's microenvironment, which have opened doors to immense developments in cardiac, neural, and bone tissue engineering [[28], [29], [30]].

The biofabrication field is a vast evolving field, of which the definition has been recently reappraised [31]. The readers are referred to several reviews for a more comprehensive overview of the historical evolution and broader meaning of the biofabrication term [[31], [32], [33]]. Similar to the biofabrication field, the tissue engineering field is also very wide. Cardiovascular diseases are the leading cause of deaths worldwide [34], with neurological disorders coming in second place [35] resulting in tremendous direct and indirect health-care costs. Additionally, bone diseases and defects are also resulting in tremendous economic and healthcare costs by severely disrupting the quality of personal and professional life [36].

Here, we review various biofabrication processes (micropatterning, fiber-based techniques, and bioprinting), that can create organized and robust tissue constructs from naturally-derived hydrogels. Their main advantages and disadvantages, as well as their recent progress and recent cardiac, neural, and bone tissue engineering applications are discussed. The challenges and potential opportunities in the field of biofabricated natural hydrogels are also outlined.

## 2 Biofabrication of natural hydrogel-based scaffolds

Mimicking the architectural features of native tissues is important in recapitulating their function with engineered tissue constructs [37]. The important aspect of controlling scaffold porosity and microarchitecture is directing tissue formation and function [38]. Scaffold porosity and pore interconnectivity affect its stiffness [39], ECM secretion [40], as well as cell survival, proliferation, and migration [41]. Original scaffold manufacturing approaches, such as solvent casting/particulate leaching, freeze-drying, and gas foaming present the ability to produce and control the size and porosity of interconnected porous structures. Yet, they do not allow the fabrication of complex geometries or controlled cellular distribution within the scaffold for developing functional and biomimetic tissues [27].

Advanced manufacturing techniques, such as microfabrication tools, fiber-based technologies, and three-dimensional (3D) bioprinting have been developed, emerging as strong tools in tissue engineering. These technologies provide a way to overcome the limitations of conventional techniques, along with allowing the precise control over mechanical properties, structural properties, microarchitecture, pore size, pore geometry, pore interconnectivity, and cellular distribution of complex engineered cell-laden scaffolds [42]. Fiber-based technologies and 3D bioprinting have been applied to a multitude of tissue engineering applications because of their robustness in creating structures with biomimetic architectures and properties enhanced by microfabrication tools [[43], [44], [45]]. In this section, we will discuss various biofabrication technologies that can be used for engineering structured constructs and scaffolds from natural hydrogels.

### 2.1 Microfabrication techniques

Numerous modern microfabrication techniques have been explored to control the microstructure of natural hydrogels to tune the cell-material interactions and cell behaviors. Photopatterning and micromolding are two of the most widely used, cutting-edge microfabrication techniques that generate 3D cell-laden hydrogel microstructures with controlled morphological, structural, and physical properties [46].

***Photopatterning***, also known as photolithography, is a technique consisting of using light to imprint patterns into materials [46]. First, a mask is created, containing the pattern to be implemented; it possesses transparent areas to pass the light and other opaque areas to block the light. Microengineered hydrogels are created via light irradiation forming the micropatterns. Areas under the transparent region of the mask are crosslinked and under the opaque parts it remains uncrosslinked; that of which are washed out afterward. Since light is used to crosslink these hydrogels, they should be photocrosslinkable. For this purpose, hydrogels can be made photocrosslinkable by conjugating acrylamide- or acrylate-based groups to the prepolymer backbone, such as in the case of gelatin methacryloyl (GelMA) [22] and methacrylate hyaluronic acid (HA) [47]. A photoinitiator is added to commence the polymerization reaction by forming radicals upon light irradiation.

Photopatterning is a flexible easy-to-use technique and allows precise spatial control over the cellular microenvironment without the need for sophisticated equipment. Furthermore, it allows the fabrication of 3D cell-laden hydrogel constructs containing various cell types by patterning different cells through sequential photopatterning. Determining the suitable ultraviolet (UV) exposure time, the fabrication of only planar constructs, and the use of multiple photomasks to control cell distribution are the main challenges faced by the photopatterning technique [48].

GelMA hydrogels were photopatterned through UV crosslinking by Nichol et al. and loaded with human umbilical vein endothelial cells (HUVECs) [49]. The results showed high cell viability after the biofabrication process and the hydrogel's mechanical properties were found to be directly affected by the UV exposure time and methacrylation degree. In a follow-up study, Aubin et al. photopatterned cell-laden GelMA hydrogel encapsulating fibroblasts, myoblasts, ECs, and cardiac stem cells with different widths to control the alignment and elongation [50]. The study proved that the widths of the photopatterned rectangular microconstructs had a significant impact on the morphology and self-organization of cells. Although UV is the most common light source for photocrosslinking of hydrogels, researchers have tried to use light sources with higher wavelengths to reduce the risk of DNA damage [51,52]. However, it should be noted that the use of higher wavelength light sources might reduce the achievable resolution.

***Micromolding*** consists of employing molds fabricated from plastics, polymers, and metals to microfabricate both physically and chemically crosslinked hydrogel constructs [48]. Micromolding is a rapid, robust, biocompatible, cost-effective, easy-to-use, and scalable technique. Most popular molds used today are fabricated from polymers such as poly(methyl methacrylate) (PMMA) and elastomers such as polydimethylsiloxane (PDMS) [53].

This technique can be used to create complex structures with high resolution via layer-by-layer fabrication and an assembly step. Layers of cationic, neutral, and anionic polymers are usually deposited onto a solid substrate, such as silica particles or sugar beads. This is well-known as the layer-by-layer technique and synonymously as electrostatic self-assembly. The multilayers are stabilized by the electrostatic forces. One or more drugs could be incorporated into the layers. Meanwhile, the layer-by-layer method has been extended to other materials such as proteins and colloids. Moreover, hollow nano and microspheres are obtained through layer-by-layer adsorption of oppositely charged polyelectrolytes on template nano- and microparticles.

**Fig. 1:**
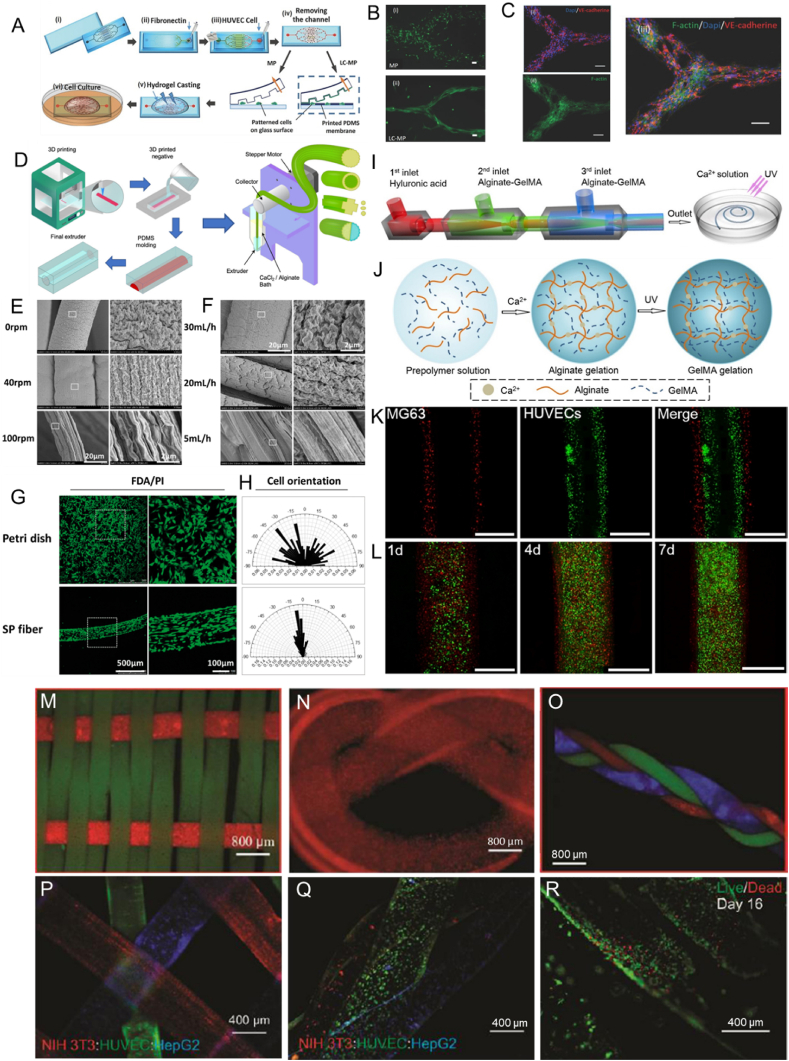
A) (i) Glass substrate and microfluidics channels are treated with oxygen plasma. (ii) Microfluidic PDMS channels are assembled on the glass substrate and fibronectin solution is loaded into the inlet port. (iii) The solution is collected, after 1 h incubation, from the outlet and meanwhile the solution containing HUVECs is loaded in the inlet. (iv) The PDMS channel is removed, after 1 h incubation, and cultured for 1 d before casting the hydrogel. (v) Hydrogel is casted on the sample. (vi) The glass substrate is then immersed in the cell media and cultured. B) Comparing the patterning quality achieved after 7 d of culture for (i) MP and (ii) LC-MP methods. C) VE-Cadherin expression (shown in red color) from HUVECs at day 11 of the culture. (i) Red (VE-Cadherin) and blue (cell nucleus) channels. (ii) Green channel showing actin filaments. (iv) Merge of red, blue, and green channels. The scale bars are showing 100 μm. Reproduced with permission [75]. Copyright 2016, Wiley-VCH GmbH. D) Schematic illustration of the fabrication process of the grooved extruders, the wetspinning device, and the hydrogel solid and hollow grooved fibers. Reproduced with permission [67]. Copyright 2020, American Chemical Society. Wetspun shear-patterned alginate hydrogel microfibers: SEM Images of the orientation trend of submicron topography on hydrogel microfibers fabricated with (E) different rotation rate ω (rpm) of receiving pool and (F) different perfusion rate Q (ml/h) of alginate, and (G) the spreading and (H) orientation of PC12 cells that were cultured on a petri dish and a shear-patterned fiber (SP fiber) after 3 days. Reproduced with permission [68]. Copyright 2017, Oxford University Press. Microfluidic fabrication of a GelMA-alginate composite natural hydrogel: (I) Ca-alginate reaction and UV exposure resulting in the formation of microfibers, (J) Schematic illustration of network formation of the composite natural hydrogel, (K) fluorescent image of cell distributions in the microfibers (HUVECs stained with CM-FDA (green) encapsulating in middle layer and MG63 stained with CM-DIL (red) encapsulating in outer layer) and (L) Confocal images of cell-laden alginate-GelMA composite hydrogel microfibers after incubation for 1, 4, and 7 days. Reproduced with permission [72]. Copyright 2016, Elsevier. Micrographs showing: (M woven alginate:GelMA fibers, (N) knitted alginate:gelatin fibers, and (O) braided alginate:GelMA fibers. Micrographs showing: (P) a multilayer construct form from different stained cell types (NIH-3T3-red; HUVEC-green; HepG2-blue), (Q) three different cell-laden alginate:GelMA fibers forming a braided cell-laden structure, and (R) high cellular viability after 16 days of culture in the braided fibers. Reproduced with permission [74]. Copyright 2015, Wiley-VCH GmbH.

In one study, a liver-like structure containing perfusable channels was created by He et al. from HepG2 cells encapsulated within micromolded agarose hydrogel; a natural hydrogel with good biocompatibility and mechanical properties [54]. Negative PDMS molds, injected with a collagen solution containing HUVECs, were used to fabricate this liver construct that had high cellular viability over 3 days of culture *in vitro*. Despite the many advantages, the fabrication of 3D vascularized geometries is not possible without the combination of other fabrication techniques, such as microfluidic systems. Rezaei Nejad et al. combined microfluidic patterning and surface micromolding techniques (Fig. 1A) for engineering high-resolution vascular-like patterns by creating planar multiscale protein, hydrogel, and cellular patterns, and simultaneously generating microscale topographical features that laterally confine the patterned cells and direct cellular growth in cell permissive hydrogels (Fig. 1B and C). Besides, the restriction to planar structures and the reduction in feature quality at high height to width ratios are two other major drawbacks of the micromolding technique.

Generally, micromolding is remarkably flexible and arguably the simplest microfabrication technique for hydrogels. On the other hand, photopatterning has rapidly evolved into a very powerful and flexible tool with many reported variations. There is still a need to incorporate and control the release of growth factors within hydrogels at predefined patterns; to stimulate cellular growth, proliferation, healing, and cellular differentiation of embedded cells. This is a problem that could be solved by microfabricating cell-laden hydrogels that are nanofunctionalized, preferably with soft nanoparticles [19].

Future developments in electroconductive composite hydrogels can lead to their fabrication using micropatterning techniques to better mimic the native ECM [55]. Moreover, advanced micropatterning techniques can be used to influence tissue function by guiding cellular interactions. Future 2D modeling systems that closely emulate human physiological functions could be developed through the micropatterning of electroconductive composite hydrogels surfaces, which holds great potential for the development of new therapeutics, stimuli-responsive systems, smart drug delivery systems, and biosensors.

### 2.2 Fiber-based technologies

Fibrous scaffolds offer anisotropic mechanical and architectural properties, which mimics those observed in some native tissues such as tendons, ligaments, and muscles [56,57]. These scaffolds can be fabricated using several different techniques such as random, organized stacking of fibers, or assembly using textile processes. Regardless of the assembly process, fibers are the essential component of such scaffolds. There exist only a few fabrication technologies that allow the engineering of continuous fibers from hydrogels including interfacial complexation, wet spinning, and microfluidic spinning.

***Interfacial complexation*** consists of fabricating fibers at the interface of two oppositely charged polyelectrolyte solutions through polyion complex (PIC) formation [58,59]. Forceps or a bent needle were used to fabricate fibers, of 10–20 μm diameters with a range of tensile strength of 20–200 MPa, by drawing upwards the contact interface of two oppositely charged polyelectrolyte droplets placed in close proximity [60]. The main advantage of this technique is that it produces fibers at room temperature under aqueous conditions, which allows the encapsulation of biologics; such as cells, proteins, and the fabrication of cell-laden hydrogel fibers [60,61]. The simplicity of this technique does not make large scale production easy to achieve. In addition, the limited type of materials that can be used and the small range of the fabricated fiber diameters, similar to the diameter of a cell, are two other major drawbacks of this technique. Natural polymers, mainly cellulose, chitosan, and alginate, were used previously to create cell-laden hydrogel fibers by interfacial complexation [60,61]. In one study, Leong et al. fabricated cell-laden alginate-chitosan hydrogel fibers using interfacial polyelectrolyte complexation to create aligned and spatially defined prevascularized tissue constructs with endothelial vessels [62]. Other previous studies have shown that vascular integration of the used tissue construct with the host is successfully promoted by the creation of a preformed microvascular network within the construct [[63], [64], [65]]. However, since this method is limited in the number of materials and fibers size range, the industrial-scale production of cell-laden structures is challenging [43].

***Wet spinning*** consists of continuous extrusion of a prepolymer from a spinneret orifice into a bath containing crosslinking reagents [58,66]. Wet spinning enables the fabrication of cell-laden hydrogels with a diameter in the range of 100 μm to several millimeters. The fabricated fibers with larger diameters carry the risk for the occurrence of weak points due to improper network crosslinking in regions far from the fiber surface. The large diameter of the fibers can also limit oxygen and nutrient diffusion, negatively impacting the survival of encapsulated cells [27]. In recent work, Mirani et al. 3D printed a grooved positive mold that was used in a casting process to fabricate grooved extruders that in turn were used in a developed wetspinning device (Fig. 1 D) [67]. Sodium alginate solution was continuously extruded into a calcium chloride bath by the fabricated grooved extruders to produce grooved solid and hollow hydrogel fibers with controlled porosity, surface morphology, and cross-sectional shape. In addition to fabricating complex 3D structures using these fibers *via* textile technologies (weaving, braiding, and embroidering), the grooved fibers were able to induce alignment along the grooves of myoblasts, cardiac fibroblasts, cardiomyocytes, and glioma cells, as opposed to their random alignment on unpatterned fibers.

Yang et al. produced shear-patterned natural alginate hydrogel microfibers with aligned submicron topography [68]. Submicron topography alignment of the wetspun hydrogel microfibers was controlled by varying the rotary rate of the receiving pool and perfusion rate of the prepolymer. Rat neuron-like PC12 cells and human osteosarcoma MG63 cells were successfully cultured in the wetspun biocompatible hydrogel microfibers. The study investigated the effect of different rotation rates of the receiving pool, different perfusion rates of alginate on the fiber topography, and the effect of this topography on the cell orientation along with the fiber axis. The results showed that the bigger the rotation rate and the smaller the perfusion rate the higher the submicron topography alignment was and that the cells cultured on shear-patterned fiber (SP fiber) showed oriented distribution, unlike the random distribution of cells cultured on a petri dish (Fig. 1 D-H).

***Microfluidic spinning*** consists of creating biofibers in a microchannel by co-flowing a prepolymer and a crosslinker in a coaxial fashion [42,69]. Fibers with a variety of structures can be produced by microfluidic spinning; this includes flat fibers, spiral curls, solid cylinders, Janus structures, hollow tubes, and bamboo-like architectures using coaxial laminar flows [70]. Microfluidic spinning enables the fabrication of cell-laden hydrogels and of microfibers in a mild environment in which most natural polymers can be spun into hydrogel-based microfibers without the use of additives [71]. Multi-compartment fiber production that mimics the native tissue's heterogeneous 3D structures, usually consisting of various types of cells, is possible when using programmable microvalves. Nonetheless, this technique is relatively slow. Other limitations include the risk of nozzle clogging during production and the need for fast solidification of fiber materials [27]. The lack of suitable materials that satisfy microfluidic fabrication is another shortcoming that was targeted by Zuo et al., who developed a combination of alginate-GelMA composite hydrogel with capillary-based microfluidic technology [72]. The microfluidic-based on composite microfibers showed improved mechanical properties compared to the one based on pure alginate. It successfully mimicked blood vessel-like microtubes by encapsulating HUVECs in the middle layer and mimicked bone ECM by encapsulating human osteoblast-like cells (MG63) in the outer layer (Fig. 1 I-L). Generally, microfluidic spinning is the most suitable spinning technique for creating cell-laden hydrogels; as it offers superior control over the fiber size, shape, and overall biochemical composition [27,42,73].

After cell-laden fibers are fabricated, textile technologies such as weaving, knitting, and braiding, can be used to fabricate tissue engineering scaffolds with tailored microarchitecture to optimize different properties and cellular behavior [25,74].

***Weaving*** consists of creating fabrics and 3D constructs by interlacing two different sets of warps at right angles [58]. Weaving can create lightweight, flexible, and strong structures with controlled geometry, porosity, morphology, and strength; achievable in a less mechanically harsh process, but possess a low porosity with small pores [42,58]. Woven natural hydrogel fibers have been used to control the cellular distribution within a construct. For example, Onoe et al. assembled cell-laden hydrogel fibers to create complex constructs with a controlled cellular pattern [76]. The microfibers had a core-shell structure, where the core was composed of gelated ECM proteins encapsulating differentiated cells or somatic stem cells, while the shell was composed of Ca-alginate hydrogel. In another study, Tamayol et al. created woven constructs (Fig. 1 M) from wetspun cell/bead-laden alginate/GelMA hydrogel fibers [74]. The pure natural hydrogel fibers were able to endure the weaving fabrication process and generated a woven construct that could be handled manually. The alginate/GelMA fibers successfully encapsulated NIH-3T3 fibroblasts for 5 days.

***Knitting*** consists of forming symmetric loops and complex patterns by intertwining threads or yarns in the form of stitches [58,73]. The knitting process is highly flexible and can create complex structures having the ability to stretch using computer-aided design (CAD) systems. On the other hand, it is a complex technique, and adjusting the knitted construct properties in different directions is challenging [27,42]. Pure natural hydrogel fibers were used by Tamayol et al. to create a gelatin knot (Fig. 1 N) [74]. A fiber of alginate/gelatin was initially used to form the knot and was then treated with an EDTA solution to remove alginate from the construct.

***Braiding*** consists of forming complex structures or patterns in a cylinder or rod shape by intertwining three or more fiber strands [42,58]. Braiding offers many advantages; such as high structural integrity, high flexibility, plus high axial, in-plane, and through-plane mechanical properties [27]. As with the other techniques, there are some disadvantages as well; geometrical limitations, inherently limiting its application and creating less porous structures [27]. Akbari et al. braided three different composite fibers (CFs) that were comprised of a load-bearing core fiber, a sheath of cell-laden natural alginate, and GelMA hydrogels; containing NIH 3T3 cells, HepG2 cells, and HUVECs, respectively, as a model for the liver [73]. However, the only pure natural hydrogel fibers that were used to produce braided constructs, were fabricated by Tamayol et al. from microbead-laden alginate/GelMA hydrogel fibers (Fig. 1 O) [74]. In the same study, 3D constructs formed from separate HUVEC-, HepG2-, and NIH-3T3-laden alginate/GelMA hydrogel fibers were stacked, braided, and assessed as a model of liver tissue (Fig. 1P–R).

Textile techniques provide the ability to engineer tissue‐like structures and scaffolds with controlled microarchitecture and cellular distribution by knitting, weaving, or braiding cell-laden fibers spun from biocompatible natural hydrogels. Additionally, these biotextiles are highly porous; in turn making them permeable to growth factors, nutrients, and oxygen. Cell-laden fiber assembly is a promising technique for building complex organs by protecting cells from the immune system during their growth, proliferation, and ECM secretion. The main challenges for the use of biotextiles for tissue engineering are the automation of the process combining textile machinery and biomaterials used to create tissues and organs. The low mechanical strength of cell-laden fibers makes their processing with the harsh textile techniques difficult, as the final structure will be too fragile to be used. The latter problem can be addressed by either coating a mechanically strong core material with a cell‐laden natural hydrogel layer to create cell‐laden fibers, or by creating cell-laden fibers from nanofunctionalized cell-laden natural hydrogels with nanoparticles that reinforce their structure [19,77]. Therefore, forming stronger fibers in an automated large-scale process is a necessity if tissue engineering scaffolds will be produced using fiber-based techniques. For this, new textile machines providing control over humidity, oxygen, CO_2_ levels, environment sterility, and nutrient access to cells encapsulated in the fibers of the generated textiles are required.

Novel textile scaffolds with greater functions will be fabricated thanks to the many advances happening in intelligent materials. For example, artificial muscles can be designed from electroactive polymers and skin grafts, that can expand in response to wound swelling, can be constructed from auxetic fibers [78]. Moreover, novel textile-based tissue engineering scaffolds will be able to intelligently monitor the physiological state of cells and to electrically stimulate them due to the developments happening in the field of conductive materials [79].

### 2.3 Bioprinting

3D printing or additive manufacturing (AM) consists of producing a three-dimensional object of almost any shape or geometry by forming successive layers of material under computer control using digital model data [9,[80], [81], [82]]. 3D bioprinting is the utilization of 3D printing technology and bioinks (bioprintable materials) to fabricate complex 3D functional living tissues by combining living cells, biomaterials, and biochemicals, using several different methods with different characteristics summarized in Table 1 [[83], [84], [85], [86], [87]]. Bioprinted scaffolds can be fabricated using several different methods, each of which possesses its own advantages, disadvantages, and material demands. In general, a typical process for bioprinting 3D tissues encompasses 6 steps and in each step, a choice should be made based on the final application and the desired properties: Imaging (X-ray, computed tomography CT, magnetic resonance imaging MRI), design approach (biomimicry, self-assembly, mini-tissues), material selection (natural polymers, synthetic polymers, ECM), cell selection (differentiated cells, pluripotent stem cells, multipotent stem cells), bioprinting technique (inkjet, microextrusion, laser-assisted), and finally the application (maturation, implantation, *in vitro* testing).

**Table 1:** Comparison of inkjet, laser-assisted, and microextrusion bioprinting techniques [[83], [84], [85], [86], [87]].

	Inkjet	Laser-assisted	Stereolithography	Microextrusion
**Natural bioink**	Alginate, agarose, cellulose, collagen, fibrin, gelatin, silk fibroin	Alginate, collagen	Alginate, gelatin, hyaluronic acid, silk fibroin	Alginate, agarose, cellulose, chitin, chitosan, collagen, fibrin, gelatin, hyaluronic acid, silk fibroin
**Material viscosity**	Low (<12 mPa/s)	Low (<300 mPa/s)	Low (<5000 mPa/s)	High (>6 × 10^7^ mPa/s)
**Preparation time**	Short	Medium to long	Short to medium	Short to medium
**Resolution**	High (10–50 μm)	High (10–100 μm)	High (25–100 μm)	Medium (20–200 μm)
**Print speed**	Fast (1–10000 droplets/s)	Medium (200–1600 mm/s)	Fast	Slow (10–50 μm/s)
**Printer cost**	Low	High	Low	Medium
**Cell viability**	High (>90%)	High (>90%)	Medium (>85%)	Low (75–90%)
**Cell densities**	Medium (10^6^-10^7^ cells/mL)	High (>10^8^ cells/mL)	Medium (<10^7^ cell/mL)	High (10^8^-10^9^ cells/mL)

***Inkjet printers*** are the most widely used printers for 2D and 3D printing; they consist of delivering controlled liquid volumes to locations that have already been defined. Inkjet bioprinters are low-cost printers with high printing speeds and can enable several polymerization mechanisms. Disadvantages of inkjet bioprinting include: their limited ability to handle liquids with limiting ranges of viscosity, restriction to thin structures, applied mechanical and thermal stresses (which reduces cell viability), and they produce solutions with low cell density [27]. Inkjet printing technology has been used for several tissue engineering applications, such as bone [88,89], cartilage [90], neural [91], cardiac [92], and skin [93]. For example, Gao et al. successfully 3D inkjet bioprinted, human stem cells, and PEG-GelMA hybrid scaffold for bone and cartilage tissue engineering; which showed improved mechanical properties and precise deposition of cells [94].

**Fig. 2:**
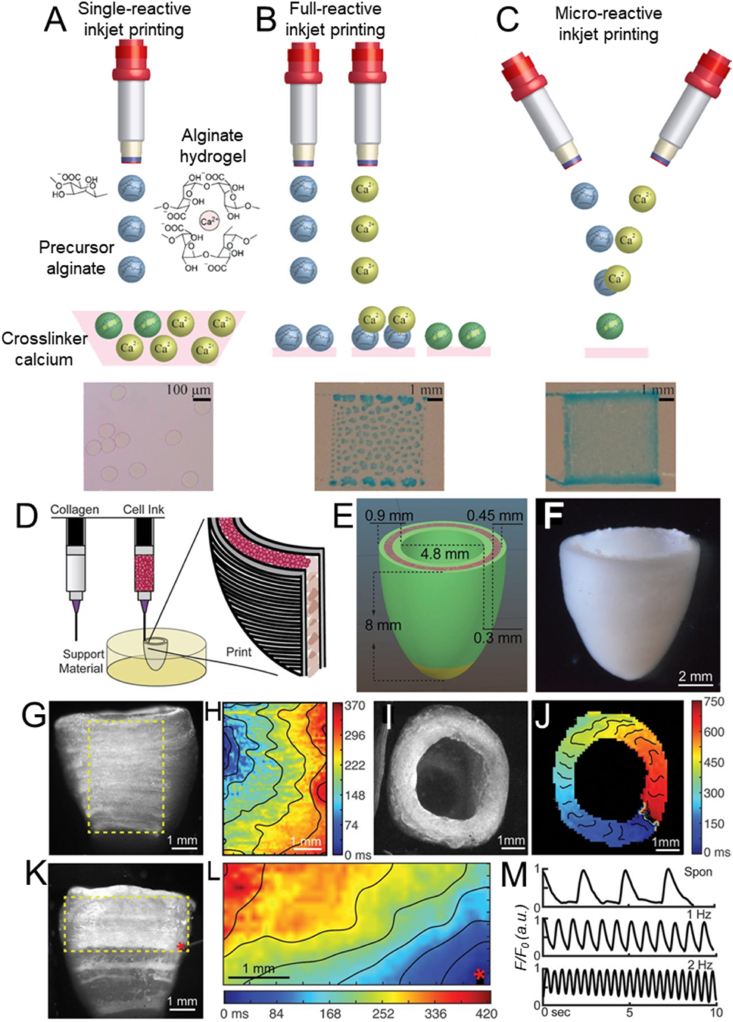
Illustration of (A) single-, (B) full-, and (C) micro-reactive inkjet printing approaches to fabricate alginate hydrogel. Reproduced with permission [95]. Copyright 2019, American Chemical Society. D) Schematic representation of the dual-material FRESH printing process and (E) the construct dimensions. F) Micrograph of the final printed model. G,I) Side and top view of the calcium imaging of the printed structure and H,J) their respective spontaneous and directional propagation calcium wave that indicates the transmission of the action potential across embedded cardiomyocytes. K,L) Calcium signal propagation observed after point stimulation. M) Transient calcium waves measured during spontaneous and induced (1–2 Hz) contractions. Reproduced with permission [96]. Copyright 2019, AAAS.

Recently, Teo et al. have created fine 3D microstructured alginate hydrogels in a single step using a micro-reactive inkjet printing technique (Fig. 2 C), based on the in-air collision of the precursor and cross-linker microdroplets [95]. This novel technique surpasses the limitations of conventional single- and full-reactive inkjet printing techniques (Fig. 2A and B), mainly the time-consuming gel substrate preparation and the complicated cross-linker bath optimization, which opens new paths for tissue engineering by 3D bioprinting microarchitectures analogous to human tissues.

***Laser-assisted bioprinting*** is based on laser-induced forward transfer; a method that was developed for the purpose of patterning and transferring metals, of which has then been successfully applied to DNA [97], peptides [98], cells [99], and other biological materials [100]. Laser-assisted bioprinting is ideal for low viscosity materials; it facilitates high cell density and achieves a microscale resolution and high cell viability [27]. There are some disadvantages to laser-assisted bioprinting; such as high cost, complexity, limited polymerization mechanisms, narrow viscosity range, and it is restricted to thin structures [27]. Laser-assisted printing technology has been used for many tissue engineering applications, such as bone [101], cardiac [96], and skin [[102], [103], [104]]. Gruene et al. bioprinted a natural hydrogel, composed of alginate and ethylenediaminetetraacetic acid blood plasma, using laser-assisted bioprinting to study the effects of different processing parameters [105]. In another study, Koch et al. demonstrated the 3D arrangement of vital cells using laser-assisted bioprinting as multicellular grafts similar to native skin model by successfully bioprinting fibroblasts and keratinocytes embedded in collagen [103].

***Stereolithography (SLA)*** consists of using light to crosslink a photosensitive resin in high-resolution patterns in the polymerization plane [32]. The main advantages of an SLA bioprinting system, are its selective solidification in a layer-by-layer manner pf photocrosslinkable hydrogels, reproducibility, high speed, high resolution that relies on the size of each micromirror, and high cell viability [106]. However, this system is only limited to photocrosslinkable polymers and to a small viscosity range. Moreover, this system usually relies on UV or near-UV light, which is harmful to cells and may form a risk of carcinogenesis, which is guiding research in this area to replace bioinks crosslinkable using UV-light with visible-light-crosslinkable bioinks. Therefore, Wang et al. investigated the SLA bioprinting of GelMA mixed with eosin Y, which is a green-light sensitive photoinitiator (514 nm), instead of Irgacure 2959 (257 nm) which is a UV-light sensitive photoinitiator [106]. In this study, they showed that only 0.02 mM eosin Y mixed with 15 w/v% GelMA resulted in a good cell viability (>80%) of the encapsulated NIH-3T3 cells that were able to not just survive and proliferate, but also to form 3D intercellular networks following their SLA bioprinting process. Digital light processing (DLP) bioprinters, which use a light projection system instead of a laser source, can print high resolutions (~1 μm) at a very fast speed (1 mm^3^/s) without nozzle system which can lead to higher cellular viabilities compared to other 3D bioprinters. This method was used by Na et al. to bioprint NIH 3T3 cell-laden silk fibroin-GelMA constructs with improved cell dispersion and in the absence of cell sedimentation compared to GelMA control constructs [107]. To prove the biofunctionality of such technique, Hong et al. successfully differentiated chondrocytes laden in bioprinted silk fibroin constructs cartilage after 4 weeks of culture [108].

***Microextrusion bioprinting*** consists of continuously dispensing biological materials and biomaterials through nozzles connected to bioink cartridges and is composed of a temperature-controlled dispensing system, a fiber-optic light source, a video camera, and a piezoelectric humidifier. Microextrusion technology has been used for several tissue engineering applications, such as bone [109,110], cartilage [111,112], neural [113,114], cardiac [115,116], skin [117,118], liver [119,120], and skeletal muscle [111,121]. Microextrusion bioprinting can create thick vertical structures, facilitate high viscosity and high cell density solutions, and enable several polymerization mechanisms; however, it possesses a tendency of nozzle clogging, achieving interlayer bonding is challenging, and in high-resolution structures, the nozzle shear can reduce cell viability [27]. Using this bioprinting technique, Billiet et al. biofabricated 3D printed macroporous cell-laden GelMA constructs for a tissue engineering application and succeeded in maintaining high cell viability (>97%) [122]. Nanocellulose–alginate bioink was successfully utilized by microextrusion bioprinters to fabricate human chondrocyte-laden natural hydrogels that maintained high cell viability and proliferation during *in vitro* culture [123,124]. This shows that the nanocellulose-alginate bioink has the potential of being used for articular cartilage tissue engineering.

Switching from bioprinting onto solid substrates to support baths has enabled the creation of more complex structures without the need to modify the biomaterial's composition to enable printability. For this, Spencer et al. 3D bioprinted cell-laden GelMA/poly(3,4-ethylenedioxythiophene):poly(styrenesulfonate) (PEDOT:PSS) bioink into a coagulation bath containing aqueous calcium chloride [125]. PEDOT:PSS was first crosslinked with bivalent calcium ions, then GelMA was photocrosslinked with a visible light, which resulted in bioprinted constructs having tunable mechanical properties, a high cytocompatibility, and a good *in vivo* biodegradability without inducing any inflammatory responses.

Recently, microextrusion bioprinting in a support bath has also been used by Lee et al. to enable the biofabrication of collagen, the primary component of the extracellular matrix in the human body, in a novel method called FRESH (freeform reversible embedding of suspended hydrogels) [96]. FRESH is based on the extrusion of bioinks within a gelatin-based thermoreversible support bath that be flushed away at 37 °C. A human cardiac left ventricle, presenting contractile functions and synchronous electroconductivity, could be bioprinted (Fig. 2D–M) using FRESH from a cardiomyocytes‐laden fibrinogen bioink confined in a collagen ink. Lee et al. successfully bioprinted five components of the human heart spanning capillary to a full-organ scale using the FRESH method [96].

An advancement in the creation of free-standing structures without the need of support materials was made recently by Connell et al. who performed *in situ* rapid photocrosslinking of GelMA bioinks as it is being extruded from the nozzle [126]. They also demonstrated a co-axial extrusion system, allowing the encapsulation of a soft or liquid core within the rapidly photocrosslinked shell filament.

Additionally, Castilho et al. developed a novel extrusion-based method called electrowriting to fabricate 3D constructs with increased resolution using gelatin- and silk fibroin-based bioinks. They managed to bioprint cell-laden fibers with a diameter as small as 5 μm into various organized shapes, while maintaining high cell viabilities, thereby providing a novel approach for bioprinting of highly detailed biofabricated constructs [127].

### 2.4 4D bioprinting

Lately, “time” is added as a fourth dimension to further develop the bioprinting technology from 3D bioprinting to 4D bioprinting [128,129]. The instances where external stimuli, such as cell fusion or self-assembly are present, cause objects to change their shape. 4D bioprinting promotes dynamic, structural, and cellular changes of tissue over time [130]. This will help overcome the static nature of 3D bioprinting and create tissue-like models that resemble the ones found in nature.

Generally, there is still not a clear classification or definition of 4D bioprinting, but two main approaches can be considered. The first approach is based on the deformation of bioprinted materials. These materials are responsive biocompatible materials, comparatively like natural hydrogels, that are able to change their shape or function according to external stimuli (i.e. water absorption, thermal stimulation, pH value, light, surface tension, and cell traction) [131]. Kirillova et al. fabricated hollow self-folding tubes by bioprinting shape-morphing cell-laden hydrogels composed of two natural biopolymers; alginate, and hyaluronic acid [132]. In this case, 4D bioprinting provided a unique control over the diameters and architectures of these self-folding tubes. The average internal tube diameter was as low as 20 μm, comparable to the diameters of the smallest blood vessels. These tubes maintained high viability of the printed marrow stromal cells for 7 days. Additionally, Kim et al. developed a photocurable silk-fibroin based bioink for DLP-based 4D bioprinting. They manufactured biomimetic trachea using this approach. These were implanted in rabbits and showed integration with the host trachea after 8 weeks [133].

The second 4D bioprinting approach is based on the maturation of engineered tissue constructs that can be achieved by cell coating, cell self-organization, and matrix deposition. For example, vascular graft maturation can be promoted by coating an endothelial cell layer in the lumen of bioprinted engineered vascular grafts to prevent thrombosis [134,135]. Similarly, a connective tubular graft was formed from the cell self-organization of printed cellular toroids that contained human ovarian granulosa cells [136]. Likewise, matrix deposition from hMSCs seeded onto bioprinted tissue constructs prolonged its degradation time from 2 days to more than 2 weeks in media [137].

In general, 4D bioprinting presents the advantages of creating 3D complex tissue constructs based on responsive biomaterials and generating tissue constructs with functionalities similar to those of native tissues [128,131]. Unfortunately, the presence of a stimulus can be a possible limitation of 4D bioprinting because it may damage or kill living cells [130]. Thus, the stimulus must be regulated or refined to prevent this problem from occurring to a significant degree.

Even though the bioprinting field is still in its early stages, it has successfully created several 3D functional living human constructs with mechanical and biological properties suitable for transplantation. Bioprinting technology can be used to print almost all types of biomaterials into scaffolds with tailored morphological, physical, and biological properties. These tailored properties can mimic native tissue properties and provide the required microenvironment for cells to grow, proliferate, and differentiate. Furthermore, patient-specific tissues can be bioprinted thanks to advancements in computer-aided manufacturing (CAM), medical imaging, and computer-aided design (CAD). Despite the wide range of advantages bioprinting present there still exists the inherent need for vast improvements and advancements in bioprinter technologies and processes.

Most bioprinting techniques still operates at a relatively low process speed; it takes hours to fabricate one construct because most bioprinters use a relatively low resolution to cover a large area. The recently introduced volumetric bioprinting technique that is based on visible light optical tomography is one of the promising exceptions [138]. Biomaterials used in bioprinting processes are in gel or sol-gel form and not in solid form; such as in the case of 3D printers, bioprinters still lack full automation [139]. Currently, most of the available hydrogel-based bioink materials, besides decellularized extracellular matrix based bioinks, lack the native characteristics present in native tissues and organs that exhibit a heterocellular architecture. A high concentration of hydrogels used in bioinks will result in a high viscosity, which favors mechanical and structural integrity and allows for the bioprinting of complex shapes. However, at the same time, these bioinks will not support cell viability and proliferation. The solution here can be the use of small concentrations of nanofunctionalized hydrogels possessing improved mechanical properties in a bioink formulation or to use a dual-step crosslinking approach when possible [140]. In terms of bioprinters commercially available on the market today, they have a high cost and limited variety.

Overall, there is vast room for improvements to be made in regard to bioprinters and bioinks; the range of compatible bioinks must be extended by using nanofunctionalized hydrogel-based bioinks, the methods of bioinks and cells deposition printed with increased precision and specificity must be developed and automated, the speed and resolution of fabrication must be increased, and finally, the high cost and size of bioprinters must be decreased.

## 3 Tissue engineering applications

Using hydrogel scaffolds for tissue engineering applications is highly advantageous, as they offer attractive characteristics for multiple applications. For example, hydrogels offer a range of mechanical properties, including desired stiffness and porosity, and allow for the incorporation of cells and bioactive molecules. Especially natural hydrogels appeal to tissue engineering applications, as they allow for improved cell adhesion, degradation of the material *in vivo* due to proteolytic activity, and exhibit inherent biocompatibility and lack cytotoxicity. Biocompatibility and biodegradability are of high importance, as adverse reactions to cells and tissue could lead to severe complications upon clinical use [141]. Moreover, the degradation rate of the scaffold should match the regeneration rate of the tissue, as this would allow for the most beneficial healing [142]. The hydrogel scaffold acts as the ECM and can integrate various growth factor hubs, specific to the tissue application, to allow for the most effective regeneration, and mimics the tissues' natural environment. It is therefore important that the mechanical properties of the scaffold match those of the inherent tissue [141,143]. Moreover, by using natural polymers, its natural structure and molecules, including RGD sequences and bioactive peptides, remain intact, which improves cell functionality, proliferation, differentiation, angiogenesis, amongst other beneficial effects [144]. In addition, biofabrication techniques using these natural hydrogels could allow for beneficial structures by mimicking the organization of the tissue, as well as improved incorporation of cells into the scaffold [145]. Moreover, the incorporation of various (nano)materials to tune the suitability and functionalize the scaffold to specific applications has been extensively examined [19]. The versatile properties of various biopolymers, including cellulose, gelatin, alginate, hyaluronic acid, and collagen, have been exploited by many researchers [[146], [147], [148]]. This chapter reviews the most recent advances in tissue engineering application using biofabricated natural hydrogels for the heart, nerves, and bone, summarized in Table 2.

**Table 2:** Tissue engineering applications of natural hydrogels in heart, nervous system, and bones.

	Hydrogel	Approach	Outcome	Ref
**Cardiac Tissue Engineering**	GelMA	Nanofunctionalization with CNTs, GO, and rGO	Good electrophysiological properties, electrical conductivity, proper mechanical stiffness, and maturation of CMs	[149]
		3D Bioprinting + Fibronectin	Enhanced CM survival and spreading	[150]
		3D Bioprinting + GNRs	Spreading of CMs and GNRs provided propagation of electrical signal	[139]
		Nanofunctionalization with GNW	Contractile behavior of CMs and enhanced maturation	[151]
	Chitosan	Nanofunctionalization with AuNPs and GO	Desirable degradation, CM maturation, increased electrical conductivity. *In vivo*, improved heartbeat and conductivity	[152]
	Collagen	Nanofunctionalization with AuNPs	Increased CM maturation, recovery of infarcted myocardium, reduced scar size	[153]
		Nanofunctionalization with CNTs	Supporting cardiac function with improved contraction	[154]
	Alginate	Injection	Promising results for cardiac regeneration	[155]
		Nanofunctionalization with peptides	Improved CM attachment and maturation and alignment	[156]
**Neural Tissue Engineering**	Collagen	Collagen tubes	Nerve regeneration in mice was observed, formation of neurites, and proper electrical behavior	[157]
	Collagen and fibrin	*In vivo* transplantation of scaffold	Enhanced axonal count	[158]
	Fibrin	Nanofunctionalization with MWCNTs and PU	Increased conductivity and neuronal regeneration	[159]
	Gelatin	Electrospinning	Schwann cell alignment and axon organization	[160]
		Electrospinning with dECM	Increased cellular function and proliferation	[161]
	Gelatin + Chitosan	Nanofunctionalization with PEDOT	Increased conductivity, neurite growth, neuronal regeneration and synapse formation	[162]
	GelMA	3D bioprinting	Cell proliferation and survival and neuronal differentiation	[163]
	Alginate	Nanofunctionalization with graphene and PVA	Increased material stiffness and electrical conductivity, PC12 cell attachment and spreading	[164]
		Nanofunctionalization with CAFGNs	Electroactive hydrogel with increased cell proliferation and improved neurite formation. *In vivo* implantation decreased inflammation	[165]
	Chitosan + HA	*In vivo* implantation	Increased formation of myelinated nerve fibers and increased myelin sheet thickness	[166]
	Silk fibroin	Electrospinning	Enhanced cell survival and neuron differentiation	[167]
		Electrospinning + Melanin	Improved signal propagation, improved cell differentiation	[168]
**Bone Tissue Engineering**	Collagen	Cryostructed porous scaffold	Mimicking of bone ECM with attachment of hMSCs. *In vivo* implantation showed promising results for meniscus regeneration	[169]
		Functionalization with HA by freeze-drying	Gradient mimicked bone structure and showed good bone functionality, osteogenic differentiation, and ALP activity	[170]
	Gelatin	Functionalization with BMP-2 and HA	Increased cell proliferation, osteogenic differentiation, and ALP activity	[171]
	GelMA	Nanofunctionalization with AuNPs and 3D bioprinting	Cell attachment, osteogenic potential, calcium deposition, ALP activity, enhanced X-ray attenuation	[172]
		3D bioprinting	High cell viability, calcium deposition, osteogenic gene expression	[173]
		3D Bioprinting and Nanofunctionalization with VEGF and silicate nanoplatelets	Mimicking of blood vessel with HUVEC incorporation. Osteogenesis and calcium deposition, as well as osteogenic gene expression	[174]
	Alginate	Nanofunctionalization with RGD-sequences	Promotion of osteogenesis and osteogenic differentiation of MSCs	[175]
	Silk fibroin	Calcium phosphate Nanofunctionalization	Self-healing properties promoting osteogenesis and formation of new bone tissue after 8 weeks *in vivo*.	[176]

### 3.1 Cardiac tissue engineering

**Fig. 3:**
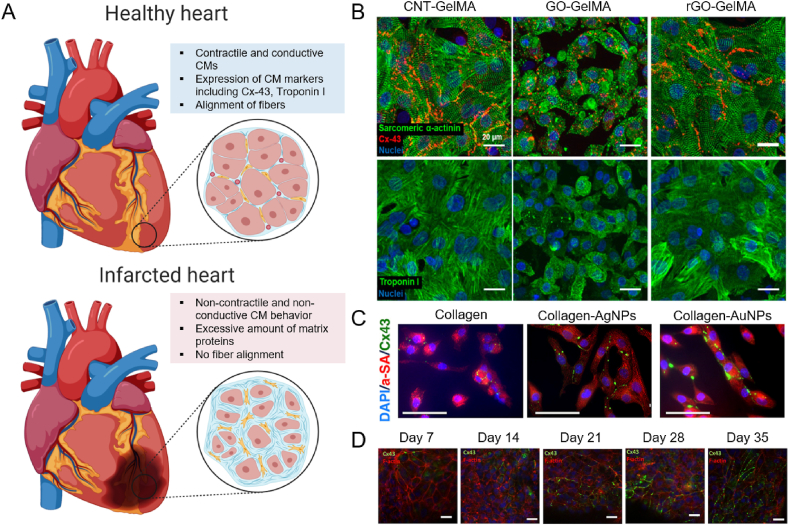
Applications of natural hydrogels in cardiac tissue engineering. A) Schematic representation of healthy cardiac tissue vs. infarcted cardiac tissue. It can be seen the infarcted region does not exhibit contractile nor conductive behavior, as a result of an excessive amount of matrix. Created with BioRender.com B) Carbon functionalized GelMA constructs, exhibiting increased Cx-43 and Troponin I expression, as well as improved cellular alignment. Reproduced with permission [149]. Copyright 2019, American Chemical Society. C) Collagen hydrogels functionalized with AuNPs shows the best expression of cardiac maturation marker Cx-43 and improved cellular alignment. Reproduced with permission [153]. Copyright 2018, American Chemical Society. D) Alginate hydrogel functionalized with RGD and HBP peptides shows increasing Cx-43 presence over the course of 35 days. Reproduced with permission [156]. Copyright 2019, Wiley-VCH GmbH.

Cardiovascular diseases, including acute myocardial infarcts, are the leading cause of death worldwide. Interruption of the blood flow in the heart leads to ischemia, which causes severe damage to the heart tissue, including loss of cardiomyocytes (CMs). Moreover, infarcts initiate the wound healing response, which induces several morphological and functional changes to the heart tissue, including excessive matrix deposition, rendering the tissue non-functional (Fig. 3 A). The infarcted region in the heart can lead to severe complications and reduced quality of life. Despite recent efforts in stem cell therapy, growth factors therapy, and extracellular vesicle delivery, that are currently being tested in pre-clinical and clinical trials, there is no fully approved cure for repairing the damaged cardiac tissue other than organ transplantation, as of yet. Engineering cardiac tissues for tissue regeneration is an emerging therapeutic strategy. Especially using natural hydrogels, cardiomyocytes or stem cells can be grown on a cardiac patch, which can be implanted onto the infarcted site of the heart, which could possibly lead to amelioration of the disease [177,178].

There are several factors that should be considered when engineering cardiac patches or cardiac tissue. In addition to being biocompatible and biodegradable, the scaffolds for cardiac tissue engineering should be able to mimic and transduce the heartbeat. This encompasses a contractible and conducive biomaterial, with according mechanical and electrophysiological properties [179]. As the heart has a low inherent regenerative capacity, it is of high importance to allow for maturation and differentiation of implanted cells into conductive and contractile CMs [180]. Therefore, the most recent advances in cardiac tissue engineering have employed nanofunctionalization of natural hydrogels, using carbon nanotubes (CNTs), gold nanoparticles (AuNPs), or graphene and its derivatives [28].

Lee and coworkers have employed GelMA hydrogel with the incorporation of CNTs, graphene oxide (GO), and reduced GO (rGO) for cardiac tissue engineering and characterized the effects of the nanofunctionalization on cardiac functionality [149]. They postulate the importance of guiding the tissue regeneration using mechanical and electrophysiological cues derived from the carbon derivatives. All scaffolds exhibited electrophysiological properties and were conductive and posed the proper mechanical stiffness for the heart. Moreover, they found that the different types of functionalization allowed for different types of maturation of the tissue. CNTs led to a ventricular-like tissue, whereas GOs guided to atrial-like tissue, and rGO-GelMA resulted in a mixed phenotype of the CMs. This is likely a result of the integrin-mediated differentiation of the CMs, which was stimulated differently by using different carbon nanoparticles. Moreover, the engineered cardiac tissue showed similar gene expression of specific cardiac genes, such as Troponin I and Connexin-43 (Cx-43) to native cardiac tissue, as well as a similar alignment of fibers (Fig. 3 B). The use of carbon nanoparticles is highly advantageous in cardiac applications, due to its electroconductive behavior, in addition to low density, high aspect ratio, large surface area, and strong mechanical resistance. However, carbon nanoparticles pose debatable cytotoxicity [181]. For all tissue engineering applications, it is of the utmost importance to perform proper testing of toxicity and safety, and therefore cytotoxicity should be evaluated carefully [182].

Other researchers have employed the beneficial effects of GelMA as well for their cardiac constructs. Koti et al. created 3D bioprinted cardiac constructs using GelMA and optimized extruder pressure as well as UV exposure, which is necessary to crosslink GelMA into a functional hydrogel [150]. In addition, they incorporated fibronectin, a naturally occurring ECM molecule, which showed enhanced CM survival and spreading. However, there are no mentions of electrical conductivity, which could pose a serious limitation. Moreover, Zhu et al. have incorporated gold nanorods (GNRs) and created a biocompatible 3D bioprinted construct, which exhibited proper spreading and organization of CMs, as well as provided an electrically active microenvironment [139]. The GNRs improved the cell-cell interactions, by traversing the pores within the hydrogel, in addition to advancing the synchronized beating of the CMs. Last, Li et al. used gold nanowires (GNWs) in GelMA, resulting in synchronized contractile behavior, as well as ideal electrophysiological properties [151]. This allowed for enhanced maturation and differentiation of CMs, creating a promising hydrogel for cardiac constructs.

AuNPs are highly attractive nanomaterials to incorporate within natural hydrogels for cardiac tissue engineering, as they are biocompatible and induce proper electrophysiological behavior to CMs [183]. Other than its use in GelMA, AuNPs have been incorporated into chitosan [152,184] and collagen [153,185] as well. The chitosan scaffold by Saravanan et al. not only incorporated AuNPs but also used GO to functionalize the hydrogel [152]. The scaffold exhibited desirable degradation properties, support of cell attachment and maturation, no cytotoxicity, and showed an increase in electrical conductivity and signal propagation. In addition, Cx-43 levels increased upon the addition of the conductive nanomaterials. *In vivo*, the scaffold showed improved heartbeat, contractility, and conductivity, creating a clinically attractive strategy. The AuNP nanofunctionalized collagen hydrogel was developed by Hosoyama et al. and showed that by using spherical AuNPs the Cx-43 expression was increased, compared to pristine collagen and incorporation of silver nanoparticles (AgNPs) (Fig. 3 C) [153]. In addition, recovery of infarcted myocardium in neonatal mice was observed upon the use of AuNP functionalized collagen. The AuNP collagen cardiac patch showed reduced scar size *in vivo*, no sign of arrhythmias, nor displacement of the spherical AuNPs, and therefore poses a promising novel therapeutic strategy for myocardial infarction.

Other researchers have employed collagen for the creation of cardiac constructs as well. Yu et al. incorporated different amounts of CNTs in a collagen hydrogel and evaluated the cardiac function of encapsulated CMs. They found that the CNT-collagen hydrogel was able to support cardiac function, as there were an improved rhythmic contraction and desirable mechanical stiffness with respect to the pristine collagen hydrogel [154].

Alginate hydrogel lends itself well for cardiac tissue engineering applications, because of its biocompatibility and ECM resemblance [186]. Researchers have been employing alginate formulations to help cardiac regeneration, and it is one of the first biomaterials tested in clinical trials [187]. A recent prospective on clinical trials using injectable alginate for patients with heart failure showed promising results for cardiovascular health, compared to the control group, indicating its high clinical potential [155]. However, there are still limitations to the use of alginate for cardiac applications, including the difficulty of recellularization due to low cell attachment [187]. Recently, Hayoun-Neeman and coworkers have employed peptides to improve cell attachment to overcome this limitation of alginate [156]. They have incorporated the arginine-glycine-aspartate (RGD) peptide, as well as heparin-binding peptide (HBP) into the alginate hydrogel and seeded stem cell-derived CMs. They monitored the cell function for 35 days, which showed increased expression of Cx-43 and alignment (Fig. 3 D), and similar results were found for the expression of Troponin I, T, and C, indicating a matured cardiac construct.

In conclusion, biofabricated and nanofunctionalized natural hydrogels, using either conductive nanomaterials or cell adhesive peptides, pose a great potential for cardiac regeneration. However, the questionable cytotoxicity of the electro-active components could pose problems upon clinical translation. Therefore, research should focus on the optimization of biocompatibility of carbon nanotubes, for example by using coatings. This will eventually allow for the clinical application of electro-active scaffolds for cardiac tissue engineering.

### 3.2 Neural tissue engineering

**Fig. 4:**
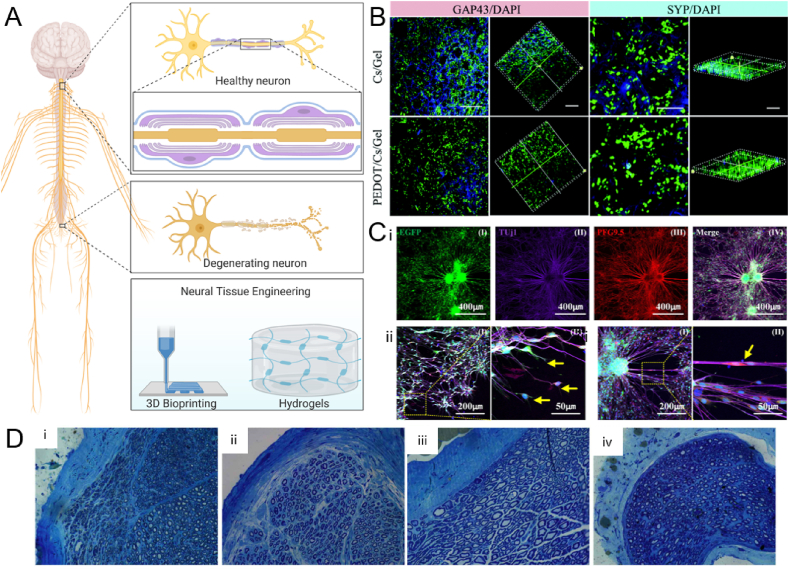
Neural tissue engineering. A) Schematic representation of the nervous system, neural degeneration, and neural tissue engineering. Created with BioRender.com B) Use of chitosan/gelatin hydrogel functionalized with PEDOT nanoparticles, enhancing the neuronal regeneration and functionality shown by neural regeneration marker GAP43 and synaptic formation marker SYP, compared with the pristine chitosan/gelatin hydrogel. Reproduced with permission [162]. Copyright 2017, The Royal Society of Chemistry. C) 3D bioprinted GelMA neuronal conduits with NCSCs, showing i) Tuj1 and PGP9.5 positive cells, indicating early neuronal differentiation ii) sprouting of neurons, indicated by yellow arrows, and iii) differentiated neural cell junction indicated by yellow arrow. Reproduced with permission [163]. Copyright 2020, Elsevier. D) Toluidine blue staining indicating myelinated neurons 12 weeks after implantation of the scaffold, showing i) chitosan, ii) chitosan/HA, iii) no implant, iv) HA, showing the most arranged and distinct axons in the chitosan/HA group, indicating the hybrid scaffold has the most optimal functionality in neural regeneration. Reproduced with permission [166]. Copyright 2018, Spandidos.

Neural tissue engineering is the repair and regeneration of the central nervous system (CNS) and the peripheral nervous system (PNS) [188]. The inherent regeneration capacity of neural tissue is very low, resulting in the requirement for external scaffolds and guidance to allow for regeneration [189]. Impairment of the neural tissue can be a result of trauma, as well as various neurodegenerative diseases including Alzheimer's Disease, Parkinson's Disease, and amyotrophic lateral sclerosis (ALS). Moreover, stroke is a large cause of brain impairment as well. These neural impairments largely decrease the quality of life of the patients and they occur frequently worldwide, resulting in great demand for the regeneration of the tissue [190]. Current strategies involve the use of (natural) hydrogels as a scaffold for neural cultures, however, biofabrication techniques are increasingly used to desirable structures to enhance neural regeneration (Fig. 4 A). Hydrogels allow transducing of mechanical cues to the neural cells, thereby mimicking the native ECM [191]. Moreover, the inclusion of magnetic particles to create a magnetic field or the addition of growth factors or conductive materials prove to be useful to enhance the functionality of the scaffold [192]. Similarly to cardiac tissue, neural tissue inherently requires electrical conductance to allow for cell-cell signaling and therefore conductive hydrogels have positive effects on the cell proliferation, differentiation, and paracrine activity, and thereby regeneration of the tissue [193]. The next paragraph discusses the most recent advances in neural tissue engineering using natural hydrogels.

Collagen is a highly used natural hydrogel for the repairment of the brain and neural regeneration, as a result of its biocompatible, biodegradable, and versatile nature. Collagen can be used as an injectable scaffold, allowing to locally deliver neuroprotective soluble factors, as well as delivery of encapsulated stem cells with regenerative capacity, or to provide structural support for the axons to grow and adhere [194]. Moreover, collagen hydrogels are one of the first hydrogels in clinical trials for peripheral nerve damage, such as CelGro (ACTRN12616001157460), Neuromaix [195], and NeuraGen [196]. O'Rourke et al. have successfully used collagen tubes combined with a neural stem cell line to regenerate a murine sciatic nerve injury model [157]. They showed the engineered tissue performed well in the regeneration of axon, the formation of neurites, and exhibited functional electrical behavior, in addition to angiogenesis. Additionally, this network outperformed autografts, which is the current golden standard treatment of neural injuries, thereby proving the functional use of stem cell lines. Schuh et al. have combined collagen with fibrin to create a functional nerve conduit [158]. *In vivo* transplantation of the scaffold into a sciatic rat model showed enhanced axonal count compared to the collagen control in the middle and distal region of the nerve.

Others have used fibrin hydrogels as well for neural tissue engineering applications, due to its excellent biocompatibility, plasticity, flexibility, ability to incorporate cells and growth factors, and because it is a component of the native ECM [197]. Hasanzadeh et al. have incorporated multiwalled-CNTs (MWCNTs) as well as polyurethane (PU) into the fibrin gel scaffold to increase its conductivity to promote neural regeneration [159]. They assessed the functionality of human endometrial stem cells and found that cell adherence, viability, and proliferation were optimal in the fibrin/PU/MWCNT hydrogel compared to the fibrin control.

Gelatin, as mentioned before, is a denatured form of collagen, which can be produced at low cost, provides high biocompatibility, and has improved cellular attachment sites, allowing for improved cellular adhesion and proliferation. In neural applications, gelatin is often electrospun, sometimes in combination with other polymers, which allows for the manipulation of functional properties. Especially the orientation of the nanofibers is easily controlled using electrospinning, ideal for neural engineering [198,199]. Electrospinning of gelatin fibers resulted in Schwann cell alignment, as well as optimal axon behavior and organization, allowing for the formation of artificial nerves [160]. Additionally, electrospinning of gelatin in combination with decellularized ECM proved high effectiveness in cellular function and proliferation [161]. However, gelatin can also be used without electrospinning. Researchers have tried to enhance the electrical properties of these gelatin scaffolds in various ways. For example, Gunasekaran et al. have created gelatin hydrogels and functionalized them with carbon black in order to create an electrically tunable scaffold, with lower electrical impedance compared to pristine gelatin [200]. Moreover, Wang et al. combined a gelatin/chitosan scaffold with PEDOT (poly(3,4-ethylenedioxythiophene)) nanoparticles, which is a biocompatible, conductive polymer, allowing for increased electrical conductivity of the scaffold compared to the non-functionalized gelatin/chitosan hydrogel [162]. In addition, cell adhesion and proliferation of PC12 neuronal rat cells, as well as neurite growth, was improved upon functionalization of the scaffold with PEDOT, shown by increased expression of GAP43, a neuronal regeneration marker, and of SYP, a synapse formation marker (Fig. 4 B).

The modification of gelatin with Methacrylic anhydride is often used for brain and neural tissue engineering as well. Rifai et al. investigated PC12 neural line's proliferation, signal propagation, and cytotoxicity when cultured on GelMA substrates, showing no sign of neurotoxic effects and good signal propagation [201]. Ye et al. have successfully implemented a 3D printing approach to create nerve guidance conduits for peripheral nerves by using GelMA and a PC12 culture [163]. They showed proper cell proliferation and survival, and in addition, the 3D bioprinted conduits allowed for neural differentiation. This was shown by culturing neural crest stem cells on the GelMA conduits and showing the abundance of an early neuron-specific marker (Tuj1), and neuron axon specific marker (PGP9.5). Enhanced green fluorescence protein (EGFP) indicates the presence of the neurons (Fig. 4 Ci). Moreover, sprouting of neurons and proper cell junctions were shown as well (Fig. 4 Cii-iii).

Alginate's pristine use has been demonstrated by Sitoci-Ficici et al., which used an alginate hydrogel system to successfully recover small spinal cord injuries, which were lesions less than 4 mm [202]. However, alginate is most often used in hybrid constructs, to improve its inherent qualities and improve the functionality of the scaffold. For example, Golafshan et al. incorporated graphene and polyvinyl alcohol to increase the toughness and electrical conductivity, which is required for nervous tissue [164]. Culture with PC12 cells showed good biocompatibility, attachment, and spreading. Homaeigohar et al. implemented citric acid-functionalized graphite nanofilaments (CAFGNs) into alginate, rendering the hydrogel electroactive [165]. The culture of PC12 on pristine alginate versus CAFGN-alginate showed increased cell proliferation and adhesion upon the addition of CAFGN, as well as improved neurite formation. In addition, *in vivo* implantation of both hydrogels in a guinea pig model showed a decrease in inflammation over the course of 2 weeks, indicating both hydrogels have good biocompatibility. Verification of improved functionality upon CAFGN addition *in vivo* is lacking. Wang et al. used a combination of chitosan and alginate for neural applications, showing the proliferation of neural stem cells, as well as olfactory ensheathing cells, showing great potential for neural tissue engineering [203]. Chitosan in combination with hyaluronic acid has been tested *in vivo* in sciatic murine models [166]. They showed an increased number of myelinated nerve fibers, increased myelin sheath thickness, and no difference in scar tissue when comparing chitosan hydrogels to HA only, chitosan only, and no treatment (Fig. 4 D). The chitosan/HA hydrogel shows great clinical potential for neural impairment and neural tissue engineering.

Silk fibroin is also often used for neural regeneration, most frequently by electrospinning it into nanofibers. The strong mechanical properties, good biocompatibility, and minimal immunogenicity allow versatile use of silk fibroin in neural tissue regeneration [190]. Li et al. have coated electrospun silk fibroin using laminin, which enhanced survival and neuron differentiation [167]. Nune et al. have employed silk fibroin electrospun fibers as well, and incorporated melanin to improve the electrical signal propagation [168]. The use of neuroblastoma cells showed good cell adherence and viability, and improved cell differentiation was observed as a result of the melanin incorporation.

In conclusion, many advances have been made towards the clinical suitability of natural hydrogels for neural tissue engineering applications. Some successful natural hydrogels, namely collagen, are already in clinical trials, highlighting the promising nature of the biomaterial. However, similar to cardiac tissue engineering, the optimal scaffold should allow for electrical signal propagation. Therefore, researchers should apply various functionalization techniques, possibly mimicking the advances in cardiac tissue engineering, to produce a conductive scaffold. Here the biocompatibility should strongly be taken into account as well.

### 3.3 Bone tissue engineering

**Fig. 5:**
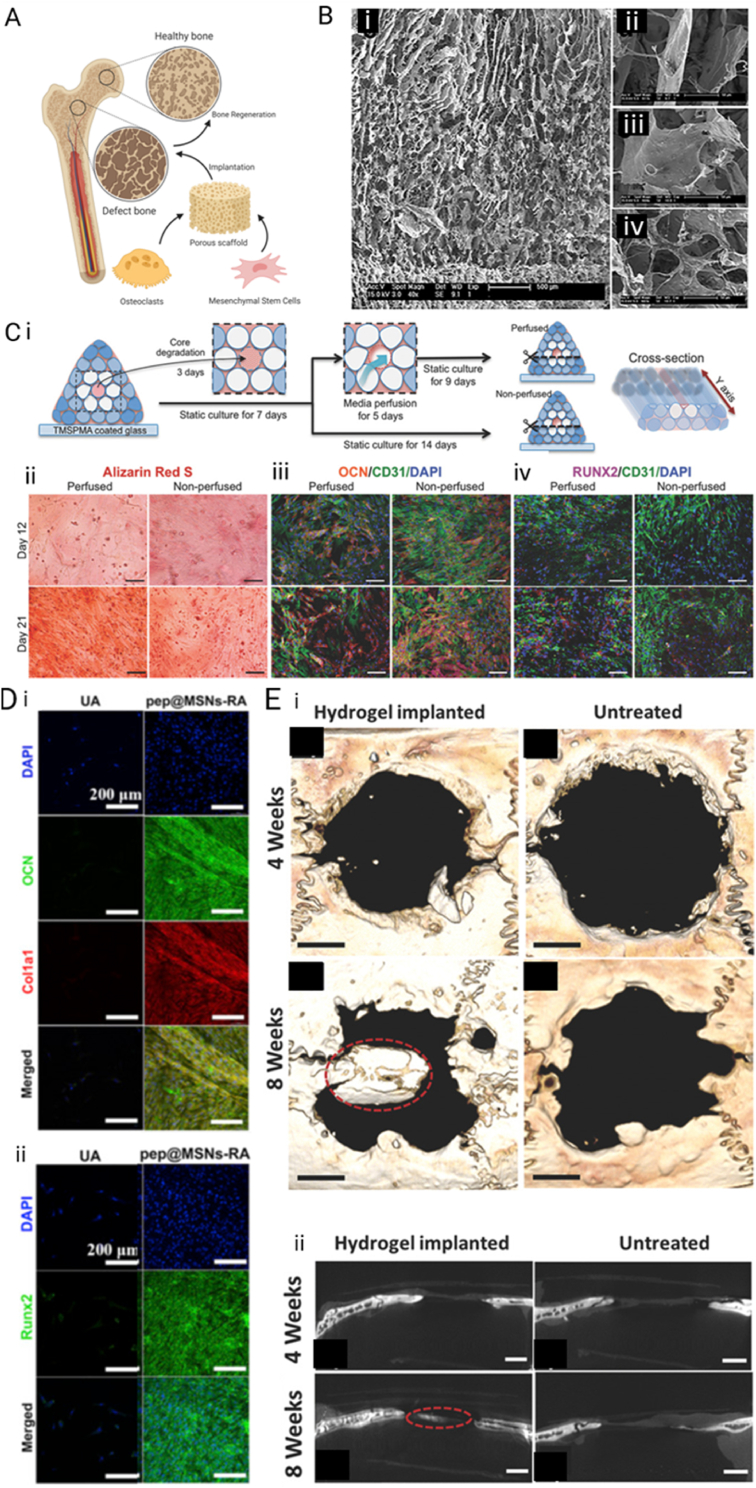
Bone Tissue Engineering applications. A) Schematic representation of defect bone and tissue engineering approach using cells in a hydrogel scaffold. Created with BioRender.com B) SEM images of bone tissue-engineered collagen scaffold with different percentage of HA functionalization. i) Overview of a scaffold, ii) 30% HA, iii) 50% HA, and iv) 70% HA. Reproduced with permission [170]. Copyright 2017, Wiley-VCH GmbH. C) Formation of 3D bioprinted scaffold for bone tissue engineering with a degradable middle vessel to incorporate HUVECs, allowing for vessel formation and perfusion, which is schematically displayed in i). ii)-iv) show increased osteogenic function in the perfused tissue by alizarin Red, OCN, and RUNX2 visualization. Reproduced with permission [174]. Copyright 2017, Wiley-VCH GmbH. D) Osteogenesis in RGD functionalized alginate gel with bone formation peptide-1 (pep@MSNs-RA), shown by i) OCN and Col1A1 immunostaining and ii) RUNX2 immunostaining, compared to control (UA). Reproduced with permission [175]. Copyright 2018, Elsevier. E) *In vivo* implantation of self-healing silk fibroin hydrogel modified with calcium phosphate and polysaccharide crosslinking showing healing of the bone in microCT analysis in i) 3D reconstruction and ii) sagittal cross-section views. Reproduced with permission [176]. Copyright 2017, Wiley-VCH GmbH.

Bone has a naturally high regenerative potential to recover small fractures and cracks, however larger bone defects, typically larger than 2 cm, cannot recover by themselves [204]. These larger defects can be a result of degenerative diseases, traumatic injuries, or surgical removal [205,206]. Bone grafts can be used to ameliorate and assist in the regeneration process of the defect bone tissue. There is a high clinical need and demand, making up an estimated cost of $5 billion in the United States alone [207]. The bone grafts that are currently in clinical use mostly comprise of autologous or allogeneic transplantation, which, in general, are biocompatible, do not invoke an immune response, and manage bone function. Especially autografts which include bone formation inducing factors, such as bone morphogenetic proteins (BMPs) or other growth factors, are of great interest. However, the limitation of such autografts is that a surgical procedure is required to harvest the tissue, and a second procedure to implant the autograft, which could all lead to very serious side effects, including defects at the donor site and other surgical risks. In addition, these procedures are highly costly [208]. Therefore, great efforts are put into research into bone tissue engineering applications of natural hydrogels, which would omit the surgical risks and donor site morbidity. It includes the creation of a biocompatible, osteogenic scaffold, which incorporates cells and in some cases growth factors to allow for optimal bone regeneration [209]. Bone tissue engineering aims to produce smart scaffolds, which sustain the dynamic nature of the microenvironment of the bone and allow for remodeling, in addition to the primary goal of bone regeneration. The scaffold replaces the bone matrix and should therefore mimic the properties of bone ECM, which include cell attachment sites, growth factor hubs, and strong mechanical properties (Fig. 5 A) [210]. In the next paragraph, the most promising applications of natural hydrogels and their functionalization for bone tissue engineering applications are outlined.

Collagen is one of the main protein components of the bone ECM and therefore lends itself well for bone tissue engineering applications, in addition to being biodegradable and biocompatible. Moreover, it contains many naturally occurring RGD sequences, which allow for optimal cell attachment [211]. One of the first examples of bone tissue engineering used a collagen hydrogel to regenerate nasal bone tissue [212]. Stuckensen et al. cryostructured collagen hydrogel precursors, resulting in a porous scaffold mimicking native bone ECM, without the use of additional growth factors [169]. hMSCs could easily enter and attach to the matrix and maintained metabolic activity. Moreover, *in vivo* implantation of the scaffold in mice showed promising results for the regeneration of the meniscus. In addition, bone tissue consists of hydroxyapatite (HA) for about 70%, the combination of collagen functionalized with HA forms a highly advantageous scaffold for bone applications [[213], [214], [215]]. Many researchers have exploited the hybrid collagen-HA scaffold as a scaffold to culture bone marrow cells [216], and more importantly to ameliorate bone defects [170]. Chen et al. produced a collagen scaffold, which contained a gradual layer of HA, fabricated using the freeze-drying process in layers (Fig. 5 B) [170]. This gradient mimicked the natural structure of bone tissue and showed increased cell proliferation, as well as native bone functionality, including osteogenic differentiation and alkaline phosphatase activity (ALP).

As gelatin is a processed form of collagen, it poses similar advantages for bone tissue engineering, including the cell attaching RGD sequences. However, as it is easily degradable and does not exhibit strong mechanical properties which are required for the bone, modifications are necessary [217]. Therefore Grazia Raucci et al. have crosslinked gelatin with carbodiimide and functionalized it with HA nanoparticles as well as BMP-2 peptide attachment [171]. They characterized the cell response to both bioactive components and found HA increases cell proliferation and induces osteogenic differentiation of hMSCs, quantified by ALP expression. Moreover, the BMP-2 peptide addition showed even higher values of ALP, indicating both promising candidates for bone tissue regeneration. Another well-known modification of gelatin is GelMA. The use of different concentrations of GelMA for bone tissue engineering was characterized by Celikkin et al. [172]. They showed the cell attachment and osteogenic potential of hMSCs through DNA content, ALP activity, and calcium deposition over the course of 4 weeks and found 5% GelMA was superior to 10% GelMA, as a result of its high porosity and pore size, high mass swelling, and other mechanical characteristics. Moreover, as X-ray is a highly used tool to visualize bone tissue, X-ray attenuation can be an important design characteristic for clinical applications. Therefore, Celikkin et al. have functionalized GelMA with gold nanoparticles and 3D printed scaffolds which enhanced the X-ray attenuation without compromising the biological activity and osteogenic potential of the scaffold [172]. Others have synthesized a highly methacrylated GelMA, exhibiting strong mechanical properties, elastic behavior, and low degradations rates, which are used as bioinks for bone tissue engineering [173]. They showed high cell viability and function, including attachment, as well as osteogenic behavior, shown by the ALP activity, calcium deposition, and expression of osteogenic genes. Byambaa et al. bioprinted 5% GelMA hydrogels as well for bone applications, which contained a hollow HUVEC (human umbilical vein endothelial cells) filled vessel, allowing to perfuse the bone tissue by mimicking a blood vessel [174]. To improve their design, they conjugated silicate nanoplatelets to the GelMA to facilitate osteogenesis, and they added VEGF (Vascular Endothelial Growth Factor) in different concentrations to promote vascularization (Fig. 5 Ci). The alizarin red staining showed high osteogenesis of calcium deposition by hMSCs (Fig. 5 Cii), as well as increased expression of osteogenic marker OCN (Fig. 5 Ciii) and RUNX2 (Fig. 5 Civ). Moreover, this proves the proliferation and cell survival over 21 days *in vitro*.

Alginate poses great possibilities for bone tissue engineering as well, however, requires some modifications to improve its suitability. To start, alginate does not exhibit the RGD sequences such as gelatin and collagen, and therefore the addition of RGD peptides opts for the promotion of cell attachment, which in addition allows for various micropatterning techniques. Moreover, alginate lends itself well to the addition of bone minerals, such as calcium, ideal for bone tissue [210]. For example, Luo et al. incorporated RGD sequences into alginate hydrogels in addition to bone-forming peptide-1 to promote osteogenesis [175]. They encapsulated hMSCs into the hydrogel and showed good proliferation of hMSCs, as well as osteogenic differentiation, shown by expression of OCN, Col1A1, and RUNX2, compared to the alginate hydrogel without RGD sequence and bone-forming peptide-1 (Fig. 5 D).

Another commonly used natural hydrogel for bone tissue engineering is silk fibroin, however, it has some limitations such as difficulty controlling gelation and crosslinking, and unsuitability for bioprinting. Therefore, researchers have made efforts in modifying silk fibroin to enhance its applicability for bone tissue engineering. Shi et al. developed a silk fibroin hydrogel, which can assemble under physiological conditions with the inclusion of a polysaccharide binder, which enables self-healing properties of the hydrogel [176]. Moreover, calcium phosphate is incorporated into the self-healing silk fibroin hydrogel to promote osteogenesis. This was shown by implantation *in vivo* and the formation of new bone tissue was observed after 8 weeks (Fig. 5 E).

All in all, promising results have been obtained for bone tissue engineering using natural hydrogels. The use of bone-specific growth factors induces increased bone formation, rendering a highly successful scaffold. For example, the silk fibroin scaffold is already able to induce bone tissue regrowth after 2 months, which should be tested over a longer period of time to notice the long term effects. Additionally, clinical trials should be conducted in order to push forward to clinical translation of the proper biomaterials, as many steps have already been taken by *in vivo* implantation and characterization of the response.

## 4 Conclusions and outlook

Novel applications focusing on the biofabrication of cardiac, neural, or bone tissues, require the design of advanced biocompatible and biodegradable scaffolds that can mimic the complex architecture of native tissues. Here, the choice of biomaterial used in the biofabrication of such scaffolds is critical. Natural hydrogels have been widely used for tissue engineering applications due to their biocompatibility and biodegradability, but more importantly to avoid the risk of inflammatory and immunological responses that can be caused by synthetic hydrogels [19].

The advances in biofabrication techniques, specifically fiber-based techniques and bioprinting techniques, hold great promise in the fields of tissue engineering and regenerative medicine. Biofabrication techniques, of which each of them offers a set of advantages and suffers from limitations when it comes to engineering complex tissues, have been discussed in this review. Biotextiles are better suited for engineering load-bearing tissues but not for very complex architectures. Whereas 3D bioprinting offers superior control over the structure's architecture and resolution but cannot produce load-bearing structures. Interestingly, 4D bioprinting is one of the emerging biofabrication technique that is highly promising for the fabrication of tubular organs (blood vessels and glands) but still requires optimization to increase the array of bioprinted biomaterials and their printing resolution, and to tune the after-folding shape and its mechanical properties.

There are two important challenges, that have been overlooked in the literature, negatively impacting the translation of biofabricated scaffolds: 1) quality control measures and defects induced throughout the fabrication process; 2) the implantation of biofabricated scaffolds. Biofabrication tools are susceptible to defects and it is probable that scaffolds contain defects which deviates from the intended architecture, mechanical properties, homogeneity of composition, etc. These defects can affect the scaffolds performance and create bottlenecks in receiving regulatory approvals for their clinical use [218]. The identification of proper quality control measures is an emerging area.

Hydrogel scaffolds are not suturable and thus their implantation *in vivo* has remained a major challenge. The use of biocompatible adhesive materials in biofabrication of scaffolds can potentially address some of the challenges. In addition, recently the concept of *in vivo* printing has emerged as a promising strategy that allows direct formation of a scaffold within the host body. So far both extrusion-based [219,220] and stereolithography-based [221] approaches have been used for *in vivo* printing of scaffolds. Upon the use of proper hydrogels and their *in situ* crosslinking, the scaffolds adhere to the surrounding tissue without the need for suturing.

The emerging applications of biofabricated natural hydrogels in cardiac, neural, and bone tissue engineering have also been discussed in this review. For cardiac regeneration, engineering electro-active scaffolds by embedding carbon nanotubes, gold nanoparticles, or graphene and its derivatives in natural hydrogels such as alginate, collagen, or gelatin can pave the way towards clinical applications. The advances in electro-active scaffolds for cardiac tissue engineering can find applications in neural regeneration since neural tissues also require scaffolds that allow electrical signal propagation. The translation of natural biocompatible hydrogels, such as collagen, is beginning to gain pace in this field. Similar to its cardiac and neural counterparts, the bone tissue engineering field has also witnessed promising results related to the use of natural hydrogels. One flagrant example is the ability of silk fibroin scaffolds to induce bone tissue regrowth in a two-month period. However, longer-term tests and clinical trials should be undertaken before a final judgment is possible.

Moving forward, to achieve a successful translation of biofabricated hydrogel products, many challenges and hurdles remain to be solved. These challenges were recently detailed and discussed by Mandal et al. [222]. Nonetheless, this has not stopped the FDA from approving a number of marketed hydrogel-based products that are usually classed as Class I, II, or III medical devices, depending on the encapsulated drugs or bioactive compounds [20,[222], [223], [224]]. Even though a bright future stands ahead for commercialized hydrogel products, due to the recent developments in biofabrication techniques, the big challenge of engineering large-scale functional tissues and organs, which has been rightfully labeled as the “Mars mission of bioengineering” [225], is yet to be “colonized”.

## Authorship contribution statement

K. Elkhoury and M. Morsink contributed equally to this work. K. Elkhoury and M. Morsink drafted and edited the manuscript of this review. L. Sanchez-Gonzalez, A. Tamayol, E. Arab-Tehrany supervised the work and revised the manuscript. All authors read and approved the final manuscript.

## Declaration of competing interest

The authors declare no conflict of interest.
